# Minutes of the Twenty-Second Annual Meeting of the Mississippi Valley Association of Dental Surgeons

**Published:** 1866-07

**Authors:** Wm. Taft


### MINUTES OF THE TWENTY-SECOND ANNUAL MEETING OF THE MISSISSIPPI VALLEY ASSOCIATION OF DENTAL SURGEONS.

*First Day.—Morning Session.*

The Mississippi Valley Association met in accordance with adjournment February 21st, 1866, at eleven o'clock in the Amphitheatre of the Ohio Dental College.

Presiding officer Julius Cheesbrough in the chair.

Roll call, members present; Drs. A. Berry, J. Taft, Cincinnati, O. H. McCollum, Augusta, Ky. Geo. W. Keely, Oxford O. Geo. Watt, Xenia, O. H. R. Smith, Cincinnati, O. M. DeCamp, Mansfield, O. Jas. Taylor, H. A. Smith, Cincinnati, O. W. H. Shadoan, Louisville, Ky. C. W. Spalding, St. Louis, A. E. Lyman, Newton Falls, O. B. D. Wheeler, Wm. Taft, Cincinnati, O. J. Cheesbrough, Toledo, O. N. W. Williams, Xenia, H. A. Beamer, Cynthiana, Ky. Geo. H. Cushing, Chicago, Ill. H. C. Howells, Hamilton, O. H. J. McKellops, St. Louis, W. C. Duncan, Cincinnati, O. C. M. Wright, Cincinnati, O. A. S. Talbert, Lexington, Ky. J. A. McClelland, Louisville, Ky.

The President, Dr. Cheesbrough, here read his opening address.

Minutes of the previous meeting read and approved.

An interval given for the payment of Annual dues.

On motion, Drs. McKellops, H. A. Smith and J. G. Cameron were appointed a nominating committee.

The Executive committee reported the following order of business.

1st. Report of Officers.

2nd. Election of officers 10 A. M. the 22nd February.

3d. Miscellaneous business, Reports of committees in order at any interval in discussions.

4th. Essays or papers to be read on the opening of discussions.

SUBJECTS FOR DISCUSSION.

1st. Dental Materia Medica.
2nd. Diseases of the teeth. Dental Physiology and Pathology.
3rd. Filling teeth. Materials and Methods used.
4th. Dental Specialties.
5th. Mechanical dentistry. New inventions.
6th. Dental Education. Relation of perceptor and student.

G. W. Keeley,

J. P. Ulrey, } Committee.

The Committee on membership consisting of Drs. A. Berry and W. H. Shadoan, reported the following names of gentlemen for the favorable action of the Association.

W. W. Allport, Chicago Ill. S Clippinger, Madison Ind. R. L Evans, Toledo, O. C. H. Harroun, Toledo O. Geo. H. Mosher, Jackson, Mich. Jas. Armstrong, Bucyrus, O. S. M. Good, Madison, Ind. R, Corson, Middletown, O. W. R. Lilly, Circleville, O. W. H. Morgan, Nashville, Tenn. A. W. Maxwell, Gallion, O. P. J. Collins, Cincinnati, O. H Newington, Jeremiah Nelson, Cincinnati, O. N. B. Stump, Ann Arbor, Mich R. A. Alloway, Montreal, Canada. J. T. Holmes, Jackson, Miss. F. C. Eddleman, Greensburg, Ind. C R. Taft, Cincinnati, B. F. Rosson, Troy O. W. M. Hammond, Cincinnati, O. W. F. Morrill, New Albany, Ind. W. G. Redman, Louisville, Ky. D. Phillips, Springfield, O. C C. Chittenden, Madison Wis. J. H. Paine, Middletown, O. Wm. H. Woodward, Ripley, Matt. Stout, Portsmouth, O. all elected.

On motion a committee of two Drs. J. G. Cameron and W. H. Shadoan were appointed to procure the services of a reporter.

Adjourned until 2 o'clock P. M.

*Afternoon Session, 2 o'clock.*

The minutes of morning session read and approved. The committee appointed to procure the services of a reporter, reported that it was impossible to get one, there being none in reach that were not engaged. The report was accepted.

On motion the first subject for discussion was taken up.

It was urged that as the subjects for discussion were very important ones to the profession that a report be made by some one of the members. Dr. Spalding kindly consented by the urgent request of the association to report until some other arrangement could be made.

Dr. Watt opened the discussions by a beautiful and clear illustration of the method by which medicinal agents were carried to and affected diseased portions of the system.

Adjourned to meet at 9 A. M. 22nd February.

*Second Day.—Morning Session.*

The meeting was not called to order until 12 o'clock in consequence of a prolonged session of the College Association.

Minutes of previous session read and approved.

The Committee on nominations reported for officers as follows: President Geo. W. Keely, Oxford, O. Vice President W. H. Morgan, Nashville Tenn. Recording Secretary Wm. Taft, Cincinnati, O. Treasurer J. Taft, Cincinnati, O. Corresponding Secretary Wm. N. Morrison. St. Louis, Mo. Executive Committee Geo. H. Cushing, Chicago Ill. A. W. Maxwell, Galion, O. A. A. Blount, Springfield, O

Publication Committee, Geo. Watt, Xenia O. B. D. Wheeler, W. C. Duncan, Cincinnati, O.

H. J. McKellops,<br>H. A. Smith, } Com.<br>J. G. Cameron,

The association went into an election with the following result.

President Geo. W. Keely, Oxford, O. Vice President W. H. Morgan, Nashville Tenn. Recording Secretary Wm. Taft, Cincinnati, O. Coresponding Secretary Wm. N. Morrison, St. Louis, Mo. Treasurer J. Taft, Cincinnati, O. Executive Com.

Geo. H. Cushing Chicago Ill. A. W. Maxwell, Galion, A. A. Blount, Springfield, O.

Publication Committee, H. A. Smith, Cincinnati, O. B. D. Wheeler, Cincinnati, O. C. M. Wright, Cincinnati, O.

Adjourned till 2 o'clock, P. M.

*Afternoon Session, 2 o'clock P. M.*

Association called to order, President Keely in the chair.

Dr. Cushing appointed Secretary Pro tem.

On motion it was Resolved that Dr. H. J. McKellops be requested to exhibit and explain before the Society the preparations recently presented to the Dental College, by Dr. A. Preterre of Paris, France; which he kindly consented to do.

On motion the Association tendered their thanks to Dr. McKellops for interesting explanation of specimens.

The Association then took a recess of five minutes.

Association called to order at the expiration of the five minute recess.

Dr. Harroun, by request laid before the Association his method of taking impressions for cleft palate appliances. On motion a vote of thanks was tendered the Dr.

On motion C. M. Wright was appointed to make a report of further discussions.

No more business being brought before the Association the discussion on Materia Medica was resumed.

On motion, Mr Hall was afforded an opportunity to present his new blow pipe and heater.

A vote of thanks was tendered the gentleman for presentation.

Adjourned

*Evening Session, 7½.*

President Keely called the meeting to order.

Minutes read and approved.

Dr. J. Taft, presented and described to the association an apparatus for making corrundum wheels invented by Dr.

Danforth, also an automatic plugger invented and manufactured by Snow and Lewis of Buffalo, N. Y.

Dr. Shadoan presented an instrument for graduating the amount of rubber to be packed into rubber cases.

On motion Materia Medica was again continued in discussion.

On motion Drs. Geo. Watt and H. A. Smith were appointed a committee to conduct a series of experiments to determine whether arsenious acid is absorbed by the tissues of the body or the pulps of the teeth.

On motion adjourned till 9 o'clock A. M. to-morrow.

*Third Day.—Morning Session.*

Association was called to order, President Keely in the chair.

Minutes of previous session read and approved.

On motion the second subject (diseases of the teeth) was laid aside and the third, filling teeth &c., was taken up.

On motion the Secretary was instructed to record all minutes of previous meetings not recorded in the regular minute book and that he be sufficiently remunerated therefore.

Moved that the regular order of business be suspended upon the opening of the afternoon session to admit the reading of papers that may not relate properly to subjects under discussion adjourned

*Afternoon Session, 1½ P. M.*

Association met, President Keely in the chair.

Minutes of morning session read and approved.

On motion the society took up the following proposed amendment to the constitution presented by W. H. Shadoan, which was amended by Geo. H. Cushing and adopted.

*Amendment to the Constitution.*

Each individual member elected as an acting member of this association shall within one year sign the constitution and pay to the treasurer the sum of three dollars and annually thereafter the sum of one dollar, and those failing to pay their annual dues for two consecutive years shall forfeit their membership.

It shall be the duty of the corresponding secretary to send a notice of the annual meetings to each member at least one month previous to said meeting.

The following resolution was adopted, Resolved; That a committee of two be appointed by the president to examine and present to the association a revision of the membership roll, to report at the next annual meeting.

Wm. Taft and C. M. Wright were appointed a committee to carry out the foregoing resolution.

A committee of two, consisting of Drs. McClelland and J. Taft, was appointed to nominate delegates to the National Association.

On motion the Secretary was instructed to notify members comprising committees of their appointment.

The announcement by the president that reading of essays was in order Dr. H. A. Smith, read a paper entitled Minor Dentistry.

Dr. Talbert read an essay on the extraction of teeth, which lead to considerable discussion.

Resolved, That the thanks of the Society be tendered to Dr. H. A. Smith for his paper and that a copy be requested for the use of the society for publication.

The committee on nomination of delegates to the National Association reported the following names, C. W. Spalding, Geo. H. Cushing, B. F. Rosson, W. F. Merrill, W. H. Woodward, B. D. Wheeler, R. L. Evans, A. W. Maxwell, J. A McClelland, H. McCollum, C. M. Wright, W. G. Redman, C. C. Chittenden, W. H. Sedgewick, R. J. Poore.

The committee on inventions made the following report which was adopted.

The committee to examine and report on inventions and improvements in competition for the reward of the silver medals provided for at the last meeting respectfully report that they have examined a rubber graduate from Dr. W. H. Shadoan of Louisville, Ky., and an Automatic Plugger from Drs. Snow and Lewis of Buffalo, also a heater and blow pipe by Mr. Hall, which the committee was compelled to rule out on account of its being a patented article. All patented articles being prohibited.

The automatic plugger by Drs. Snow and Lewis we consider by far the best instrument of the kind that has ever come under our notice and to these having no assistant it is a valuable instrument.

The rubber graduate by Dr. Shadoan is certainly one of the most accurate and simple instruments for ascertaining the exact quantity of rubber for a piece of rubber work that we have ever seen.

A. S. Talbert, } Com.

W. H. Shadoan, }

Dr. J. A. McClelland, illustrated an original plan for a spittoon so arranged as to collect waste gold from fillings.

*Report of Treasurer.*

The following are the receipts and disbursements for the year ending this 22nd day of Feb. 1866.

J. Taft in act with the Miss. Valley Dental Association.

| | |
|---|---|
| To amount on hand at last report, | $47 68 |
| To amount of Annual dues to date, | 30 00 |
| To amount of Initiation fees to date, | 60 00 |
| | $137 68 |

Respectfully submitted,

J. Taft, Treasurer.

On motion an auditing committee consisting of Drs. Sedgewick and A. S. Talbert was appointed to examine the treasurers report.

On motion Drs. Shadoan, Mollyneaux and Cameron were appointed examining committee for inventions, improvements, &c. for the next annual meeting.

The following resolutions were adopted by the Association.

Resolved. That this association award a medal to the value of one hundred dollars for the most approved essay on alveolar abscess.

Resolved. That this association award a medal the value of one hundred dollars for the best essay on diseased antrum.

It was moved and adopted that a committee of five be appointed to examine essays, and that the committee be intrusted with power to reject all essays if deemed unworthy of a prize.

The president appointed Drs. Geo. Watt, H. A. Smith, C. M. Wright, G. W. Keely.

The committee appointed at the last meeting of the *Miss. Valley Association of Dentists* to arrange a device for a medal awarded to Dr. C. H. James, for best mechanical improvement would respectfully report: That they have had a medal prepared in silver, with suitable inscription upon it, for which they expended the sum of twenty dollars, the amount appropriated for the same. The medal has been delivered. February 21, 1866.

H. A. Smith,<br>C. M. Wright, } Com.

Resolutions offered and adopted.

Resolved. That all dental and medical journals be requested to publish the proposition of this association to award the various medals mentioned in the proceedings of this society.

Resolved. That the committee on prize essays be governed by the same rules as were adopted in reference to the essay on anæsthesia at the annual meeting two years ago, and that those rules and regulations be published in full with the announcement of the prizes now offered.

Adjourned

*Evening Session*, 7½

Meeting called to order Pres. Keely in the chair.

Minutes read and approved.

Dr. Cushing of Chicago was called upon to read a paper which he had prepared entitled "Dental education."

A vote of thanks was tendered Dr. Cushing for his essay and a copy requested for publication.

On motion the President appointed Drs. J. C. Dean, C. M. Wright, A. A. Blount, N. W. Williams, H. J. McKellops and W. H. Sedgewick to prepare essays for the next annual meeting.

On motion the fourth subject for discussion was passed over and the fifth taken up.

Resolved. That the sum of fifteen dollars ($15) be paid the Janitor of the Ohio Dental College for services during the sittings of this association.

A vote of thanks was tendered the Reporter.

A proposition of Dr. Morgan, in regard to the best essay on health of dentist, accepted.

Society adjourned to meet on first Tuesday in March 1867. at 10 o'clock, A. M.

Wm. Taft, Secretary.

